# Effects of the Combined Abdominal Draw-In Maneuver and Manual Resistance on Lumbopelvic Muscle Activity and Anterior Pelvic Tilt During Prone Hip Extension

**DOI:** 10.3390/bioengineering12111252

**Published:** 2025-11-16

**Authors:** Dong-Woo Kim, Young-Jun Shin

**Affiliations:** Department of Physical Therapy, College of Health, Kyungwoon University, Gumi-si 39160, Gyeongsangbuk-do, Republic of Korea; kdwkjh@ikw.ac.kr

**Keywords:** prone hip extension, abdominal draw-in maneuver, manual resistance, gluteus maximus, lumbopelvic stability

## Abstract

This study investigated the effects of applying the abdominal draw-in maneuver (ADIM) and manual resistance (MR), separately and in combination, during prone hip extension (PHE) on muscle activity and anterior pelvic tilt. Twenty-four healthy adult males performed PHE under three randomized conditions: ADIM, MR, and ADIM combined with MR. Electromyography was used to measure gluteus maximus (GM), erector spinae (ES), internal oblique (IO), and hamstring activity, while anterior pelvic tilt angle was assessed using a gyroscopic sensor. Repeated-measures ANOVA revealed significant differences across conditions (*p* < 0.05). Post hoc analysis showed that GM and IO activity were significantly greater in the ADIM combined with MR condition than in either ADIM or MR alone, with MR also producing higher values than ADIM (*p* < 0.05). ES activity was lowest in the ADIM condition, while ADIM combined with MR produced lower ES activity than MR (*p* < 0.05). The GM/ES ratio was highest in ADIM combined with MR compared with the other conditions (*p* < 0.05). Anterior pelvic tilt angle was significantly smaller in both the ADIM and ADIM combined with MR conditions compared with MR (*p* < 0.05). These findings suggest that combined ADIM with MR induces strong IO contraction and enhances lumbopelvic stability, leading to substantially increased GM activity.

## 1. Introduction

Prone hip extension (PHE) is widely used as a representative exercise to strengthen the gluteus maximus (GM) and to enhance lumbopelvic stability [1,2]. PHE is utilized to assess lumbopelvic stability, during which excessive lumbar extension, pelvic rotation in the sagittal or horizontal plane, GM weakness, and compensatory activity of the erector spinae (ES) and hamstrings can be observed [3]. Clinically, in individuals with lumbopelvic dysfunction, PHE often reveals excessive ES activity and reduced GM activation, resulting in hyperextension of the lumbar spine and increased anterior pelvic tilt [2,4]. Increased anterior pelvic tilt has been associated with impaired postural control, increased body sway in the frontal plane, and a higher risk of falls, partly due to weakness of the gluteal and abdominal musculature [5,6]. Repetition of such movement patterns may impose continuous stress on the facet joints, potentially leading to low back pain or movement-related injuries [7,8]. Therefore, maintaining lumbopelvic stability during hip extension is important to reduce ES overactivity and prevent excessive lumbar extension [9,10].

To achieve lumbopelvic stability and improve functional movement, strengthening of the abdominal muscles is essential [11,12]. The abdominal muscles regulate trunk motion, maintain spinal alignment during limb movement, and reduce the load on spinal joints [7,13]. In particular, weakness of the transversus abdominis (TrA) and internal oblique (IO) has been shown to increase anterior pelvic tilt and lumbar lordosis, thereby contributing to spinal instability [14,15]. Furthermore, TrA and IO weakness can lead to dominant activity of the ES, resulting in altered lumbopelvic movement patterns [16,17]. Thus, strengthening the TrA and IO is crucial for maintaining spinal neutrality and restoring segmental stability [17,18].

One of the most widely used techniques to selectively strengthen the TrA and IO is the abdominal draw-in maneuver (ADIM) [1,12]. ADIM, which involves drawing the navel toward the spine, selectively activates the TrA, IO, and multifidus [19]. This maneuver enhances spinal segmental stability and promotes coordination between global and local muscles, thereby contributing to anticipatory postural control and functional movement [20,21]. Specifically, when ADIM is applied during hip extension in the prone position, it reduces compensatory ES activity, increases GM activation, and decreases anterior pelvic tilt [4,9].

The concept of selective muscle activation is also applied in manual resistance (MR) training. MR is a useful method in the early stages of rehabilitation to strengthen weakened muscles, as the therapist provides an appropriate level of force directly to the target muscle [20]. This approach allows the patient to experience precisely directed resistance vectors and finely adjusted loading for effective muscle activation [22]. Moreover, the therapist can adjust resistance intensity and vary the point of application to minimize compensatory movement and enhance strengthening effects across different angles [16].

To date, various studies have examined selective activation of the GM during PHE. Oh et al. [4] reported that applying ADIM during PHE suppresses excessive ES activity while facilitating GM activation. Park et al. [1] found that stretching of the iliopsoas prior to ADIM further enhances GM activity during PHE. Jeon et al. [9] demonstrated that combining ADIM with knee support in a table-supported position increases selective GM activation. Although these approaches have provided valuable insights, no study has yet investigated the combined application of ADIM and MR during PHE. Therefore, the present study aimed to examine the effects of applying ADIM with MR during PHE on the activation of the GM, ES, IO, and hamstring as well as on anterior pelvic tilt angle.

## 2. Materials and Methods

### 2.1. Subjects

This study employed a single-session, within-subject, randomized crossover design. All procedures were conducted at Kyungwoon University (Gumi-si, Gyeongsangbuk-do, Republic of Korea) between 1 July and 31 September 2025. A total of 24 healthy adult male participants were recruited through bulletin board postings and announcements on the university’s LMS (Table 1). Interested individuals voluntarily contacted the research team and were enrolled after receiving detailed explanations about the purpose, methods, and safety of this study. Written informed consent was obtained from all participants prior to participation. The study protocol was approved by the Institutional Review Board of Daegu University (IRB No.: 1040621-202505-HR-038).

The required sample size was calculated based on previous studies using similar research designs [9,12]. A power analysis performed with G*Power 3.1.9.7 software, assuming an effect size of 0.3, a power of 0.8, and a significance level of 0.05 [23], indicated that a minimum of 18 subjects was required. However, to compensate for potential dropouts and to enhance the reliability and robustness of the findings, a total of 24 participants were recruited.

Inclusion criteria were healthy adults in their twenties without any orthopedic, neurological, or functional impairments of the lumbar spine, pelvis, or lower extremities. Exclusion criteria included a history of pain, deformity, or surgery in these areas, or any condition that could interfere with safe performance of the PHE.

Participants performed the PHE under three experimental conditions: (1) ADIM, (2) MR, and (3) combined ADIM + MR. The order of the three conditions was randomized and counterbalanced using the RAND() function in Microsoft Excel to minimize order effects.

### 2.2. Surface Electromyography (EMG) Recording and Data Processing

Muscle activity of the GM, ES, IO, and hamstring was recorded during the PHE using a wireless surface EMG system (TeleMyo DTS EMG, Noraxon Inc., Scottsdale, AZ, USA). Prior to electrode placement, the skin was prepared to reduce impedance: hair was shaved with a fine razor, the stratum corneum was lightly abraded with sandpaper, and the area was cleaned with alcohol swabs to remove residual oils. Disposable Ag/AgCl electrodes were then applied at standardized anatomical sites. Specifically, electrodes for the GM were positioned midway between the greater trochanter and the sacrum [24]; for the ES, 2 cm lateral to the spinous process of L1 [24]; for the hamstring, halfway between the gluteal fold and the popliteal crease [4]; and for the IO, diagonally 2 cm distal and inferior to the anterior superior iliac spine [12].

The EMG signals were collected at a sampling rate of 1500 Hz using Myo-Research Master Edition 1.06 XP software. A band-pass filter between 20–400 Hz was applied, along with a 60 Hz notch filter to remove electrical noise. The raw data were processed to root mean square (RMS) values, and muscle activity was normalized to the percentage of maximal voluntary isometric contraction (%MVIC). For MVIC testing, Kendall’s manual muscle testing protocol was followed [22]. Each contraction lasted 5 s, and the mean of the middle 3 s was calculated, excluding the initial and final 1 s. Measurements for each muscle were repeated three times, and the average value was used for analysis. The GM/ES muscle activation ratio was calculated using the mean values of the collected EMG data of the GM and ES.

### 2.3. Measurement of Anterior Pelvic Tilt Angle

During the PHE task, the anterior pelvic tilt angle was assessed using a 4-D MT system (ReLive, Gimhae, Republic of Korea). The anterior pelvic tilt angle was measured concurrently with the EMG recordings to ensure synchronized data acquisition. The sensor was securely attached to the midline of the sacrum with double-sided adhesive tape in accordance with the procedure described by Oh et al. [4]. Data were continuously collected during the movement and transmitted wirelessly via Bluetooth to a tablet device. For statistical analysis, the maximum anterior pelvic tilt angle recorded during each trial was used.

### 2.4. Experimental Procedures

Before the experiment, all participants were instructed in the PHE and the ADIM and completed a 10 min familiarization session to ensure that they could perform the PHE while maintaining the ADIM correctly. During the familiarization phase, the pressure biofeedback unit (PBU; Chattanooga, TN, USA) was used to teach proper abdominal contraction in the prone position. The PBU was placed under the abdomen at the level of the umbilicus, and the baseline pressure was set at 70 mmHg. Participants were instructed to gently draw in the lower abdomen toward the spine during exhalation to maintain or slightly decrease the pressure by 5–10 mmHg. After mastering this technique, participants performed the PHE while maintaining the ADIM, ensuring that they could perform the maneuver accurately and consistently during the subsequent trials.

Participants then performed the PHE under three randomized conditions: ADIM, MR, and ADIM combined with MR. A 5 min rest period was provided between conditions to minimize muscle fatigue and learning effects. In all conditions, the dominant leg was used, with dominance determined verbally by asking which leg the participant usually used more frequently and perceived as stronger.

**Figure 1:**
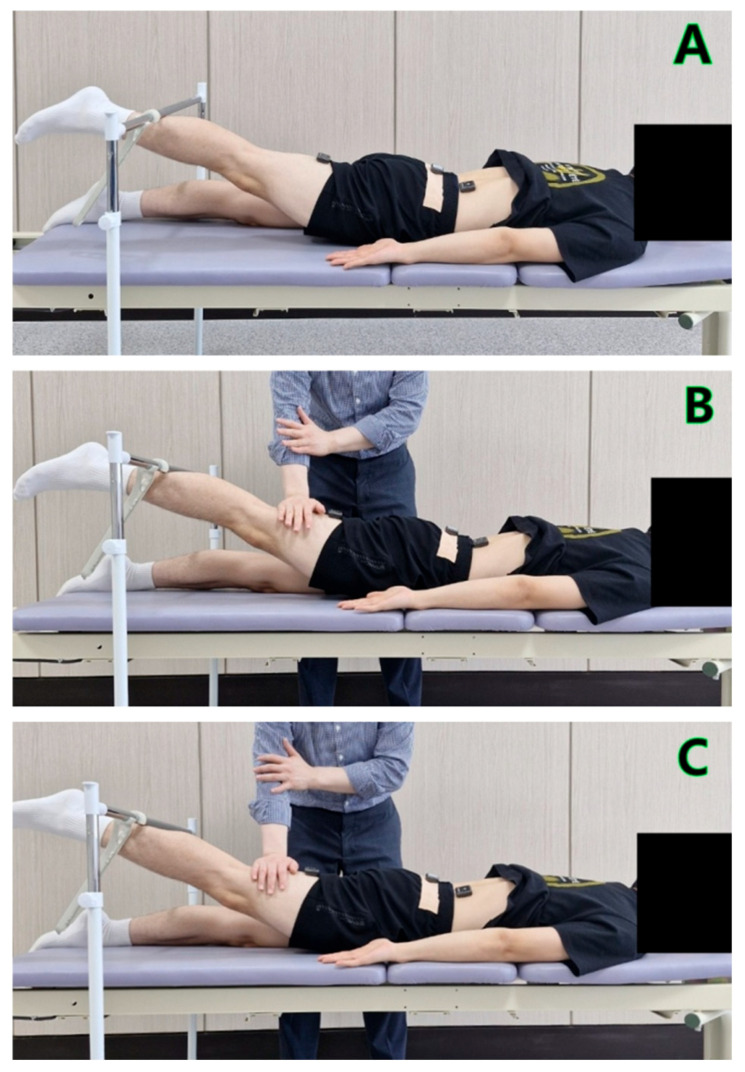
(**A**) prone hip extension with the abdominal draw-in maneuver; (**B**) prone hip extension with manual resistance; (**C**) prone hip extension with the abdominal draw-in maneuver combined with manual resistance.

In the ADIM condition, participants assumed a prone position with their arms relaxed at the sides and the dorsum of the hands facing upward to maintain anatomical alignment. The target bar was set at 10° of hip extension using a goniometer [4]. A guide bar was installed to restrict hip abduction, and any trial in which the leg contacted the guide bar was considered invalid. Upon the examiner’s verbal cue, participants performed the ADIM by drawing in the abdomen, then extended the hip with the knee straight. After the leg touched the target bar, they maintained an isometric contraction for 5 s, during which the ADIM was also maintained (Figure 1A). MR was applied to the distal posterior thigh to induce and maintain isometric contraction at the target hip extension angle of 10°. The resistance was adjusted individually according to each participant’s strength to ensure that the hip joint remained stable without visible movement. To ensure consistency across participants, the manual resistance was applied by the first author, a licensed physical therapist with 15 years of clinical experience, who followed standardized hand placement and verbal cueing procedures. Each task was repeated three times with a 30-s rest interval. EMG data were recorded during the 5 s of isometric contraction, and the mean of the middle 3 s was analyzed after excluding the first and last second. The maximum anterior pelvic tilt angle was used for analysis.

In the MR condition, participants performed the PHE without ADIM. When the leg touched the target bar, the examiner applied MR vertically against the hip extension to induce an isometric contraction (Figure 1B). All other procedures were identical to those of the ADIM condition.

In the ADIM combined with MR condition, participants performed the PHE while maintaining the ADIM, and MR was applied in the same manner as in the MR condition. The remaining procedures were the same as in the other conditions (Figure 1C).

### 2.5. Statistical Analyses

Statistical analyses were performed using PASW Statistics 18 (SPSS Inc., Chicago, IL, USA). The Shapiro–Wilk test was used to assess the normality of the data. To examine differences in EMG amplitudes of the GM, ES, IO, hamstring, and GM/ES ratio across the three conditions, a one-way repeated-measures ANOVA was conducted. When the assumption of sphericity was violated, the Greenhouse–Geisser correction was applied. Post hoc comparisons were conducted using the Bonferroni correction, with a significance level set at *p* < 0.05.

## 3. Results

Significant differences were observed in GM, ES, IO, and hamstring activation, as well as in the GM/ES ratio, during PHE across the three conditions (*p* < 0.05) (Table 2). Post hoc analysis revealed that GM activity was significantly greater in the ADIM combined with MR condition compared to both the ADIM and MR conditions (*p* < 0.05), and was also greater in the MR condition than in the ADIM condition (*p* < 0.05). ES activity was significantly lower in the ADIM condition than in both the MR and ADIM combined with MR conditions (*p* < 0.05), and was also lower in the ADIM combined with MR condition than in the MR condition (*p* < 0.05). IO activity was significantly higher in the ADIM combined with MR condition than in the other two conditions (*p* < 0.05), and was also higher in the MR condition than in the ADIM condition (*p* < 0.05). Hamstring activity was significantly greater in both the MR and ADIM combined with MR conditions compared to the ADIM condition (*p* < 0.05). Finally, the GM/ES ratio was significantly higher in the ADIM combined with MR condition than in the other two conditions (*p* < 0.05).

**Figure 2:**
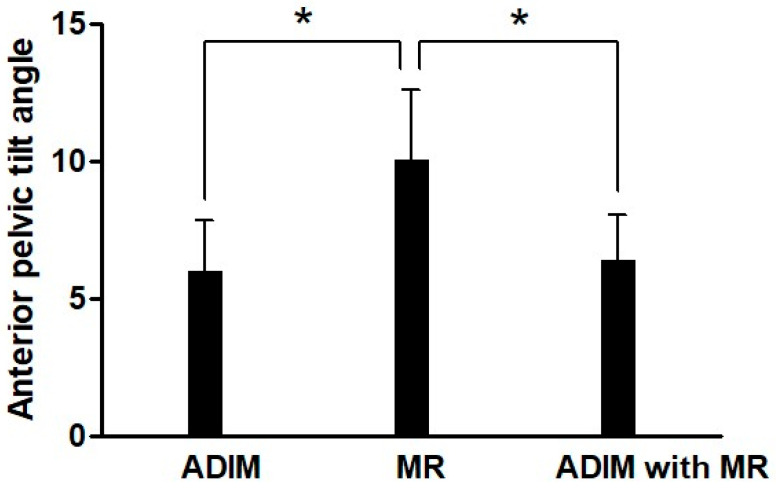
Anterior pelvic tilt angle during prone hip extension under three conditions. ADIM, abdominal draw-in maneuver; MR, manual resistance; * *p* < 0.05.

Significant differences were observed in anterior pelvic tilt angle across the three conditions (*p* = 0.001) (Figure 2). Post hoc analysis revealed that both the ADIM combined with MR and the ADIM conditions showed significantly lower values compared to the MR condition.

## 4. Discussion

The application of ADIM during PHE enhances the activation of the TrA and IO, thereby improving lumbopelvic stability, reducing ES activity, suppressing compensatory anterior pelvic tilt, and effectively promoting selective activation of the GM [4,9,25]. MR provides precise directional force and finely adjusted loads, enabling selective muscle activation and is considered an effective method for enhancing target muscle activity [16,22]. Therefore, the present study compared the effects of ADIM, MR, and their combined application during PHE on the activity of the GM, ES, IO, and hamstring, as well as on anterior pelvic tilt.

The findings of this study demonstrated that in both conditions with MR applied (MR and ADIM combined with MR), muscle activity was significantly greater across all muscles compared to the ADIM-only condition (*p* < 0.05), with notable absolute differences. Resistance training is widely recognized to promote neuromuscular adaptations by enhancing motor unit recruitment and firing rates, and by improving coordination among agonist, antagonist, and synergistic muscles [26,27]. Supporting this, Elgueta-Cancino et al. [28] reported that resistance training led to significant improvements in maximal strength as a result of increased motor unit recruitment and firing frequency. Likewise, Van Hooren et al. [29] suggested that resistance exercise lowers recruitment thresholds, accelerates firing rates, and enhances intermuscular coordination, thereby enabling more efficient management of high-intensity loads. This finding supports the view that the pronounced increase in muscle activity observed during isometric PHE against MR in this study may be explained by these underlying neuromuscular mechanisms.

The results of this study showed that the ADIM combined with MR condition produced significantly higher muscle activity of the GM and IO, as well as a greater GM/ES ratio, compared to the ADIM and MR conditions (*p* < 0.05). In addition, ES activity and anterior pelvic tilt angle were significantly lower in the ADIM combined with MR condition than in the MR condition (*p* < 0.05). These findings suggest that the combined application of ADIM and MR induced strong contractions of the IO and TrA, thereby enhancing lumbopelvic stability and simultaneously suppressing anterior pelvic tilt. As a result, ES activity increased relatively less, while GM activity, acting as a force couple with the IO in a stabilized lumbopelvic state, increased substantially. Although TrA was not directly measured in this study, its activity could be indirectly inferred through IO activation, consistent with previous studies that used IO as a proxy to indirectly measure TrA activity [30,31,32].

When comparing the ADIM combined with MR condition to the ADIM condition, ES activity was significantly increased (*p* < 0.05), whereas no significant difference was observed in anterior pelvic tilt angle (*p* > 0.05). Typically, increased ES activation during PHE is associated with greater anterior pelvic tilt [1,4,9]. In contrast, the combined application of ADIM and MR in this study appeared to enhance IO activation, which facilitated posterior pelvic tilt. Consequently, despite the increase in ES activity, changes in pelvic tilt appeared to be effectively suppressed. Moreover, the ADIM combined with MR condition elicited a significant increase in overall muscle activity compared with the ADIM condition (*p* < 0.05), and the GM/ES ratio was markedly higher (*p* < 0.05). Collectively, these findings suggest that ADIM combined with MR may serve as an effective intervention during PHE to suppress anterior pelvic tilt while concurrently promoting overall muscle activity and enhancing GM activation relative to ES.

In this study, MR was applied by adjusting the intensity in real time according to the participant’s movement, thereby inducing isometric contraction. Based on the results of this study, MR was confirmed to be an effective method for inducing more precise contraction of the target muscles, likely because it allows for real-time adjustment to individual strength levels. Cho et al. [33] reported that when ADIM was applied in combination with hip flexion resistance exercises using elastic bands in stroke patients in a sitting position, improvements were observed in balance, postural control, and functional performance. In contrast, Dafkou et al. [34] combined ADIM with barbell loading during the supine bridge exercise but did not identify significant changes in abdominal muscle thickness. These contrasting results are considered to be attributable to differences in the type of resistance applied. Specifically, elastic bands and MR, which adaptively provide resistance according to movement, appear to facilitate muscle activation and functional improvements, whereas fixed and uniform loads such as barbells appear not to be effective in promoting selective muscle activation. Therefore, it is suggested that the type and method of resistance application play an important role in determining the effects of resistance exercise when combined with ADIM.

The limitations of this study are as follows: First, since only healthy adult males were included, it is difficult to generalize the findings to patients or other age groups. Second, the intensity of MR could not be quantified. Third, as this was a cross-sectional study, only immediate effects were examined. Therefore, future research should consider differences according to age and sex, and evaluate both short-term and long-term effects in patients with lumbar or hip dysfunction and pain. In addition, it will be necessary to investigate the effects of combining ADIM and MR with other exercises. Future studies should also include participants with varying degrees of anterior pelvic tilt and use quantitative assessments such as posturography to enhance generalizability.

## 5. Conclusions

This study confirmed that during PHE, ADIM combined with MR increased GM and IO activation, improved the GM/ES ratio compared with ADIM or MR alone, and decreased anterior pelvic tilt compared with MR alone. These findings suggest that ADIM combined with MR may be a useful strategy to promote relative strengthening of GM compared with ES and to enhance lumbopelvic stability.

## Figures and Tables

**Table 1:** General characteristics of subjects (n = 24).

Characteristics	Mean ± SD
Age (year)	23.36 ± 1.81
Height (cm)	174.21 ± 3.62
Weight (kg)	67.38 ± 4.51
BMI	23.23 ± 1.28

SD, standard deviation.

**Table 2:** EMG activity of each muscle during prone hip extension under three conditions.

Muscle Activity (%MVIC)	ADIM	MR	ADIM Combined with MR	F	*p*
Gluteus maximus	22.97 ± 6.29 ^a,b^	66.91 ± 9.09 ^c^	74.77 ± 7.63	361.70	<0.001 *
Erector spinae	28.88 ± 7.34 ^a,b^	77.40 ± 8.12 ^c^	68.40 ± 8.60	263.59	<0.001 *
Internal oblique	22.87 ± 6.32 ^a,b^	40.22 ± 7.01 ^c^	57.67 ± 8.79	359.64	<0.001 *
Hamstring	39.03 ± 8.74 ^a,b^	80.54 ± 7.95	77.38 ± 8.03	236.63	<0.001 *
Gluteus maximus/ Erector spinae ratio	0.82 ± 0.33 ^b^	0.86 ± 0.16 ^c^	1.11 ± 0.18	11.22	0.001 *

Values are presented as mean ± standard deviation. MVIC, maximal voluntary isometric contraction; ADIM, abdominal draw-in maneuver; MR, manual resistance; * *p* < 0.05; ^a^ Significant difference between ADIM and MR conditions; ^b^ Significant difference between ADIM and ADIM with MR conditions; ^c^ Significant difference between MR and ADIM with MR conditions.

## Data Availability

Data supporting the reported results are not publicly available due to privacy/ethical restrictions.

## Abbreviations

The following abbreviations are used in this manuscript:

PHE	Prone Hip Extension
GM	Gluteus Maximus
ES	Erector Spinae
IO	Internal Oblique
TrA	Tranversus Abdominis
ADIM	Abdominal Draw-In Maneuver
MR	Manual Resistance
